# Vacuolar Interface Dermatitis as a Histologic Reaction Pattern of Sjögren’s Syndrome: A Case Report

**DOI:** 10.7759/cureus.46412

**Published:** 2023-10-03

**Authors:** Georges El Hasbani, Abdul-Ghani Kibbi, Ali Jawad, Imad Uthman

**Affiliations:** 1 Medicine, St. Vincent's Medical Center, Bridgeport, USA; 2 Dermatology, American University of Beirut Medical Center, Beirut, LBN; 3 Rheumatology, Royal London Hospital, London, GBR; 4 Rheumatology, American University of Beirut Medical Center, Beirut, LBN

**Keywords:** adverse side effect, autoantibodies, disease-modifying antirheumatic drugs, cutaneous manifestations, sjögren’s syndrome

## Abstract

Sjögren’s syndrome (SS) has been widely known for its dry mouth and dry eyes presentation. Extraglandular disease manifestations may be protean and pose a challenge for clinicians, especially when the typical known manifestations are absent. Skin involvement of SS is variable, and cutaneous signs and symptoms may be the initial presentation of this syndrome. Vacuolar interface dermatitis has been linked to dermatomyositis and systemic lupus erythematosus, but rarely to SS. Herein, we present the case of an 87-year-old man who presented for widespread itchy erythematous scaly plaques that were refractory to topical corticosteroids as well as discontinuation of possible offending medications. A biopsy demonstrated vacuolar interface dermatitis in the setting of strongly positive anti-SSA. Hydroxychloroquine treatment was effective in resolving the plaques.

## Introduction

In addition to the typical sicca syndrome, Sjögren’s syndrome (SS) may have a wide spectrum of extraglandular manifestations, including cutaneous involvement [1]. Common cutaneous manifestations of SS include xerodermia, cutaneous vasculitis, and erythema annular [2].

Interface dermatitis is non-specific pathologic reaction pattern characterized by vacuolization at the dermoepidermal junction, keratinocyte damage disruption of the basement membrane, and a variable lymphocytic infiltrate in the dermis inflammatory infiltrate [3]. It is observed in many skin diseases including connective tissue disease such as lupus erythematosus and dermatomyositis [4].

In this case report, we highlight vacuolar interface dermatitis as the initial presentation of SS in an 87-year-old gentleman who had no dryness of the eyes or mouth and no reported joint involvement.

## Case presentation

An 87-year-old man with past medical history of essential hypertension, diabetes mellitus, benign prostatic hyperplasia, and treated hypothyroidism presented with progressive itchy erythematous rash over the torso and extremities of one-month duration. He also reported unintentional 10 kg weight loss. Notably, he denied any dry mouth symptoms, dryness of the eyes, arthralgias, or morning stiffness. He self-treated with topical corticosteroids, topical and oral antihistamines with minimal response.

**Figure 1:**
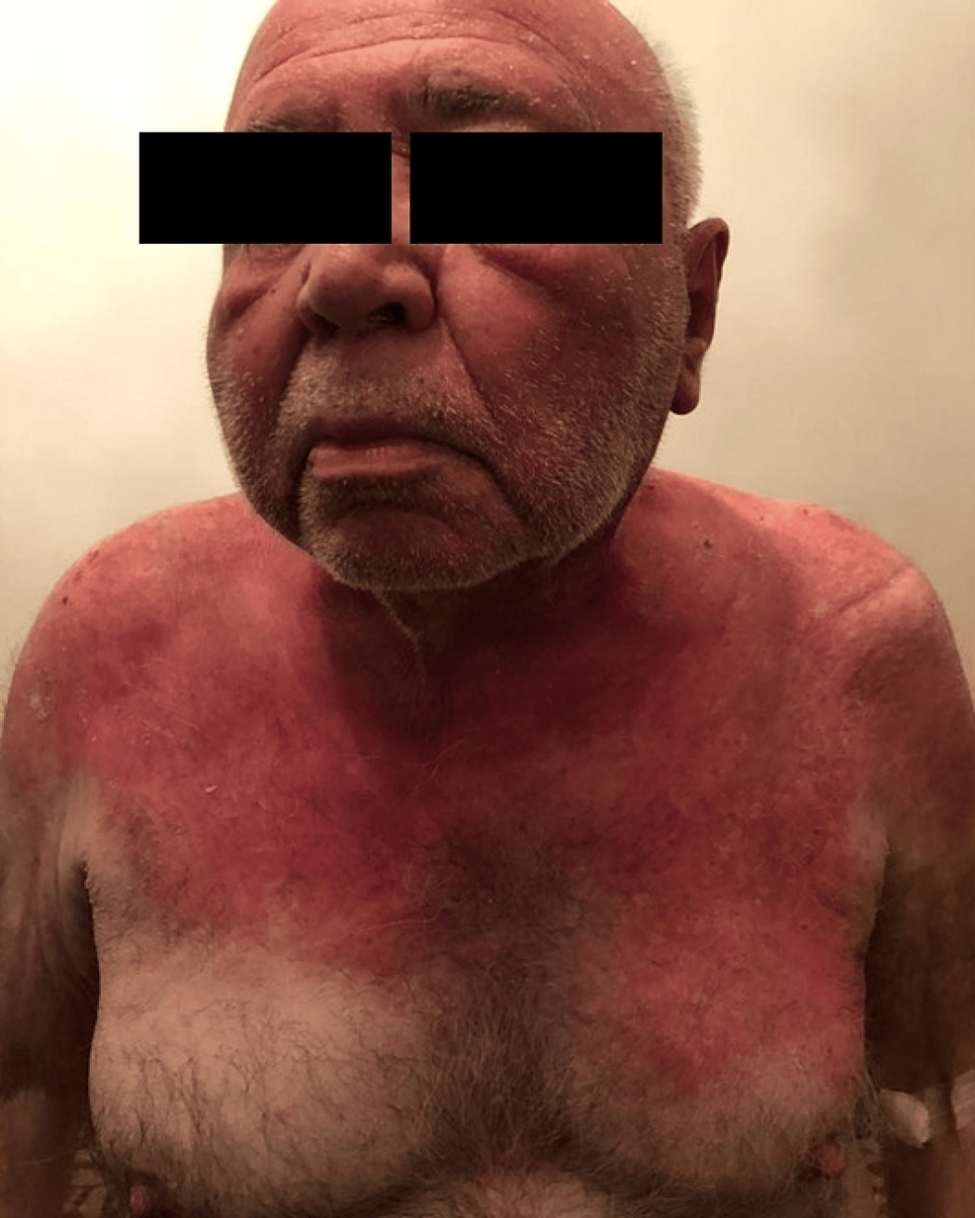
Widespread erythematous scaly plaques over the periorbital areas and chest

**Figure 2:**
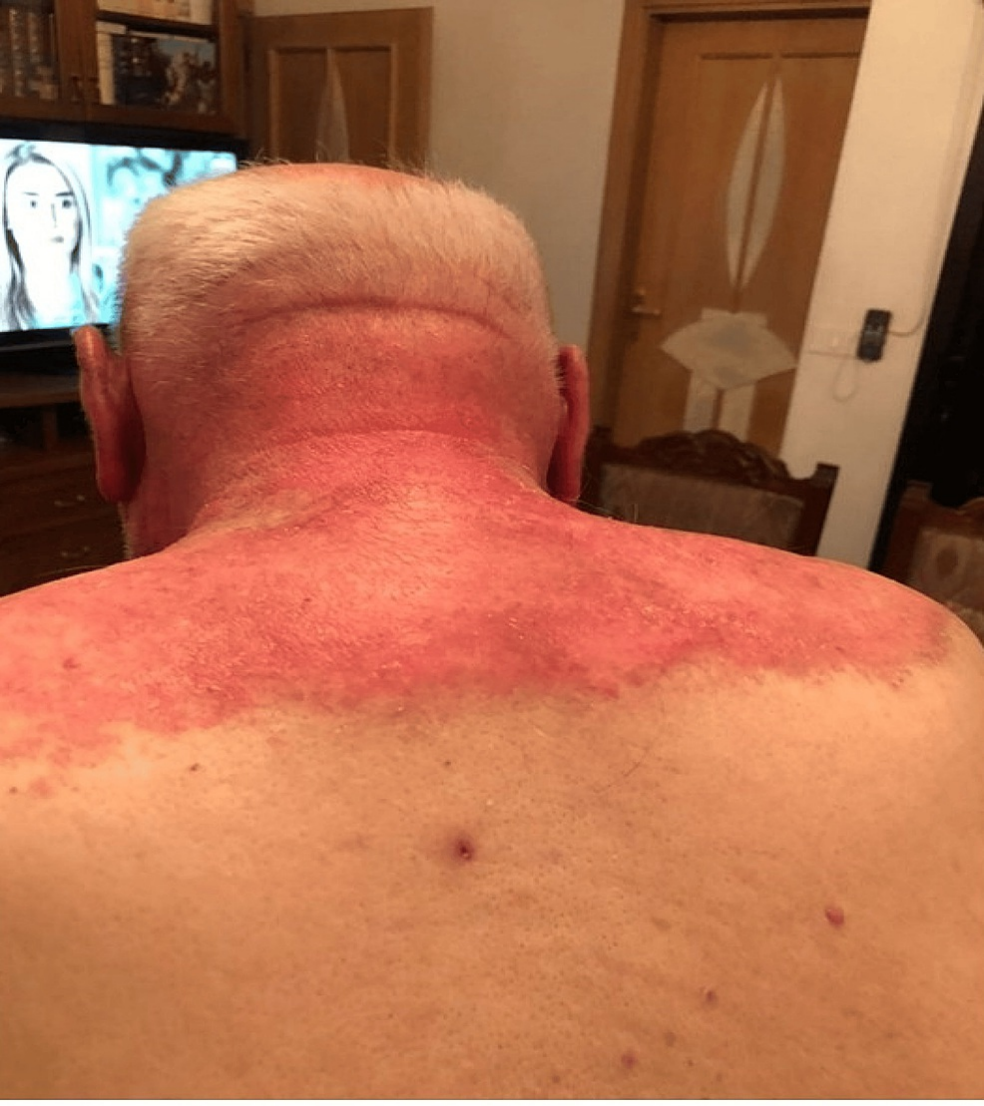
Widespread erythematous scaly plaques over the upper back

**Figure 3:**
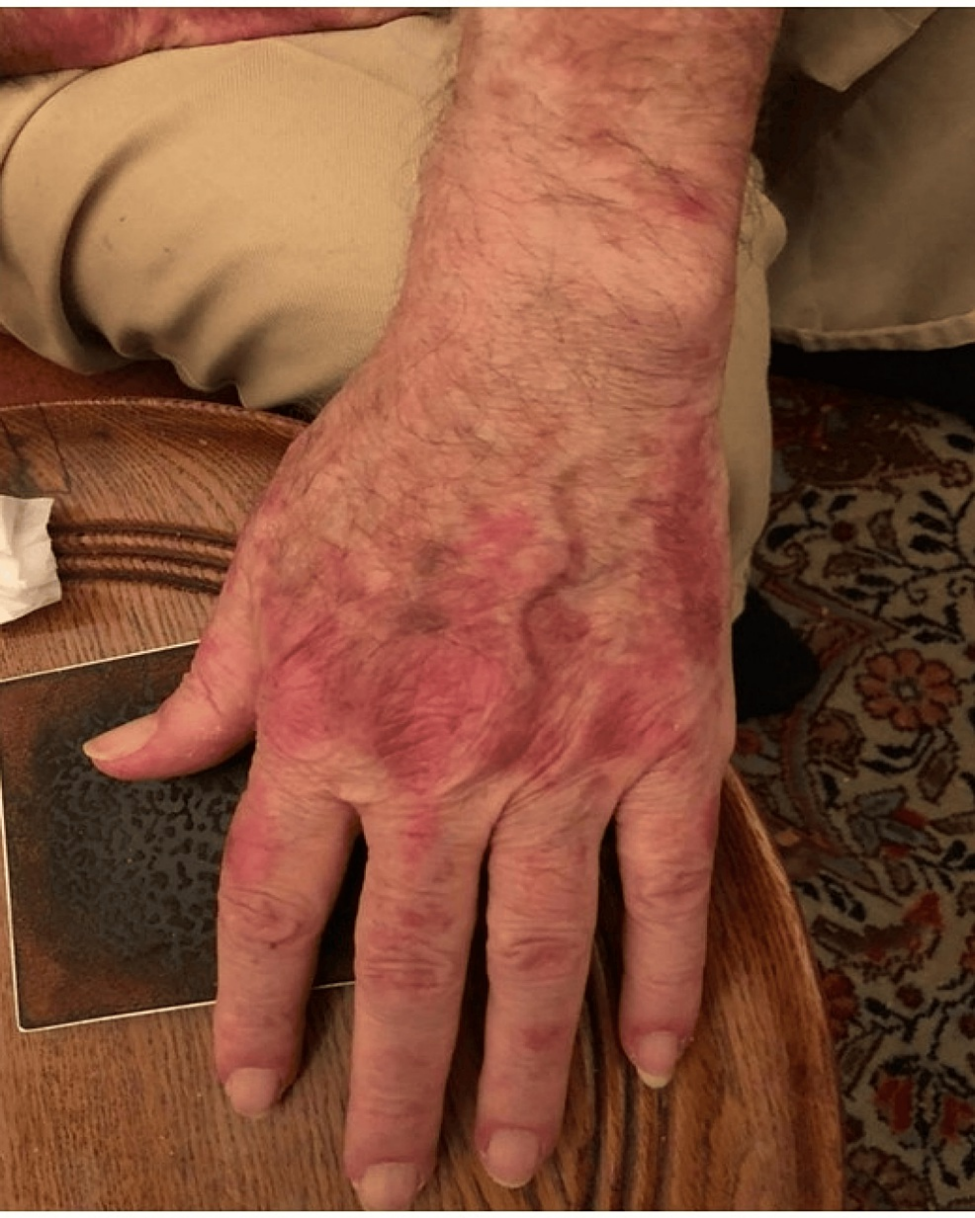
Dusky violaceous papules and patches over the dorsa of the hands

Physical examination revealed widespread erythematous scaly plaques over the periorbital areas as well as dusky violaceous papules and patches over the dorsa of the hands, palms, upper back, and chest (Figures 1, 2, 3).

**Figure 4:**
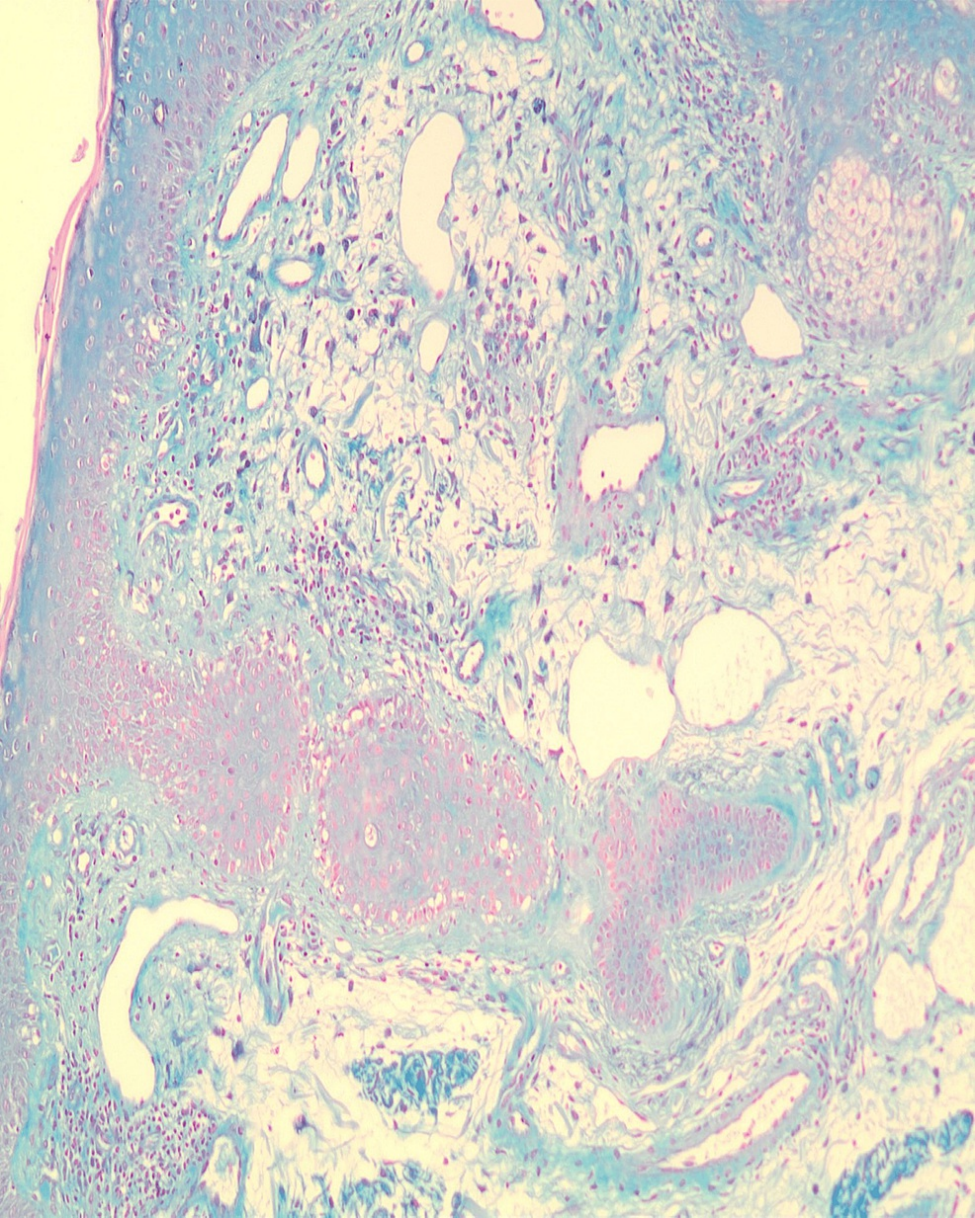
Interface dermatitis, vacuolar type in association with dermal mucinosis

**Figure 5:**
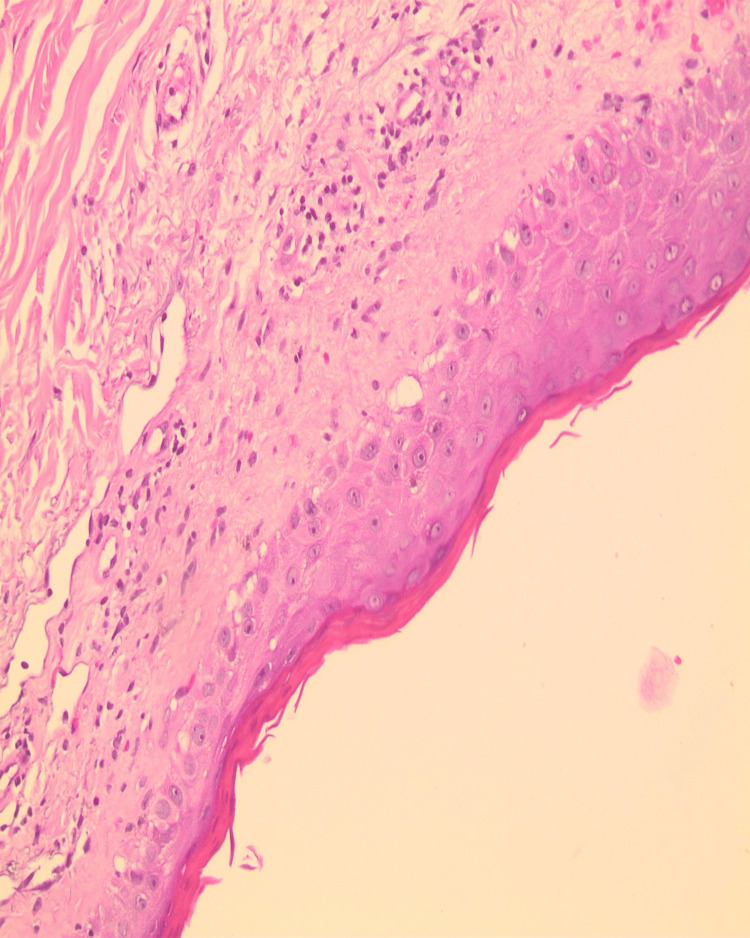
Vacuolar Interface dermatitis pattern with dermal mucinosis

Complete blood count and serum biochemistry were within normal limits. Punch biopsies from the dorsum of the hand and periorbital area demonstrated vacuolar interface dermatitis pattern of injury in association with dermal mucinosis (Figures 4, 5). Possible contributing medications, such as metformin, amlodipine, and bisoprolol were discontinued with no remarkable effect.

As the biopsy results indicated potential compatibility with either systemic lupus erythematosus (SLE) or dermatomyositis, an intramuscular corticosteroid injection was promptly administered. Subsequently, a low dose of prednisolone was initiated, following a tapering regimen. Importantly, his creatinine phosphokinase (CPK) levels remained within the normal range. An autoimmune screen showed an anti-SSA/Ro52 of 203 U/mL (Normal <10 U/mL) and anti-SSB/La antibodies of 54.8 (Normal <10 U/mL). Anti-double-stranded DNA antibodies (anti-ds-DNA antibodies) and anti-Smith antibodies were negative.

**Figure 6:**
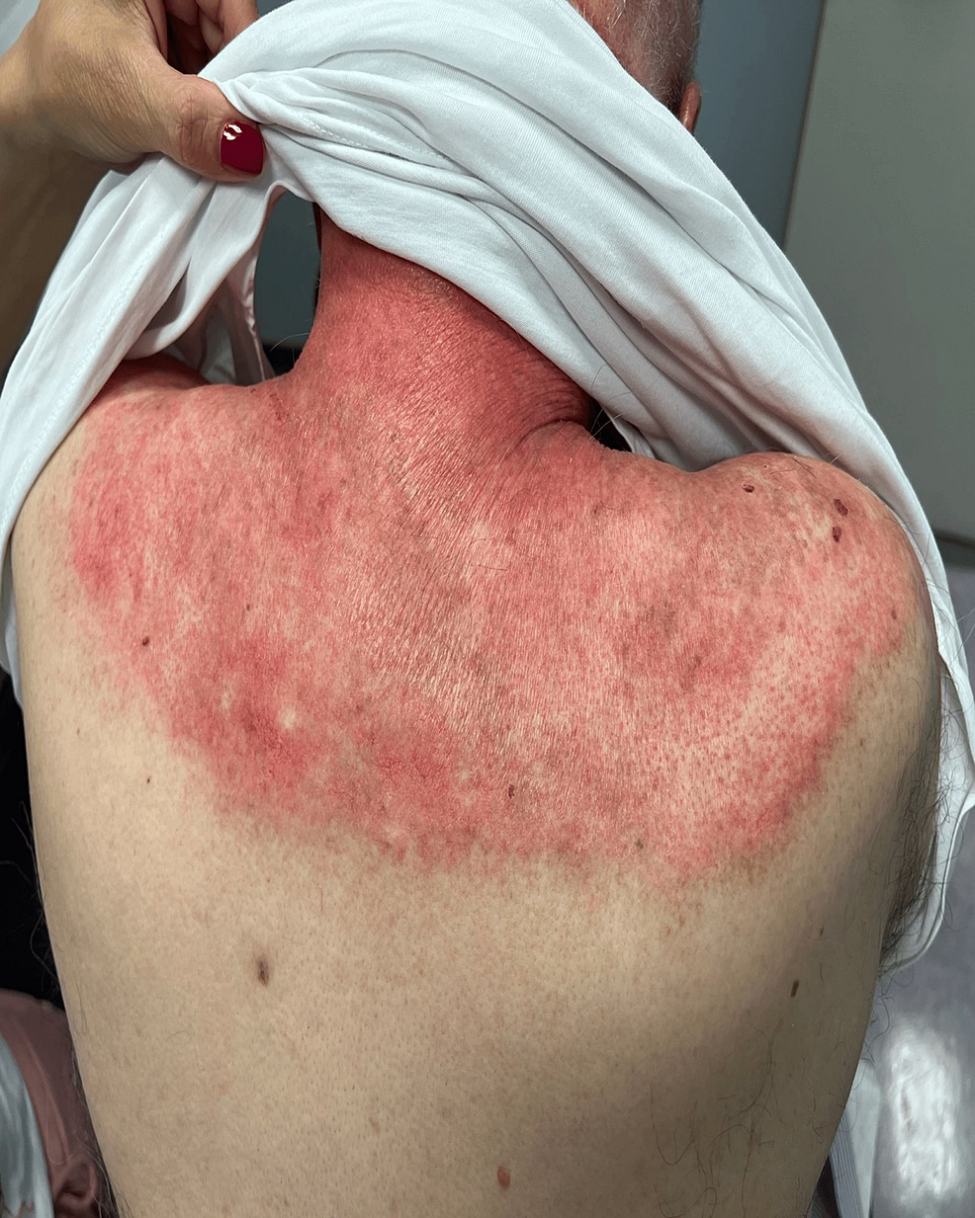
Erythematous scaly plaques over the upper back a few weeks after initiation of treatment with hydroxychloroquine

**Figure 7:**
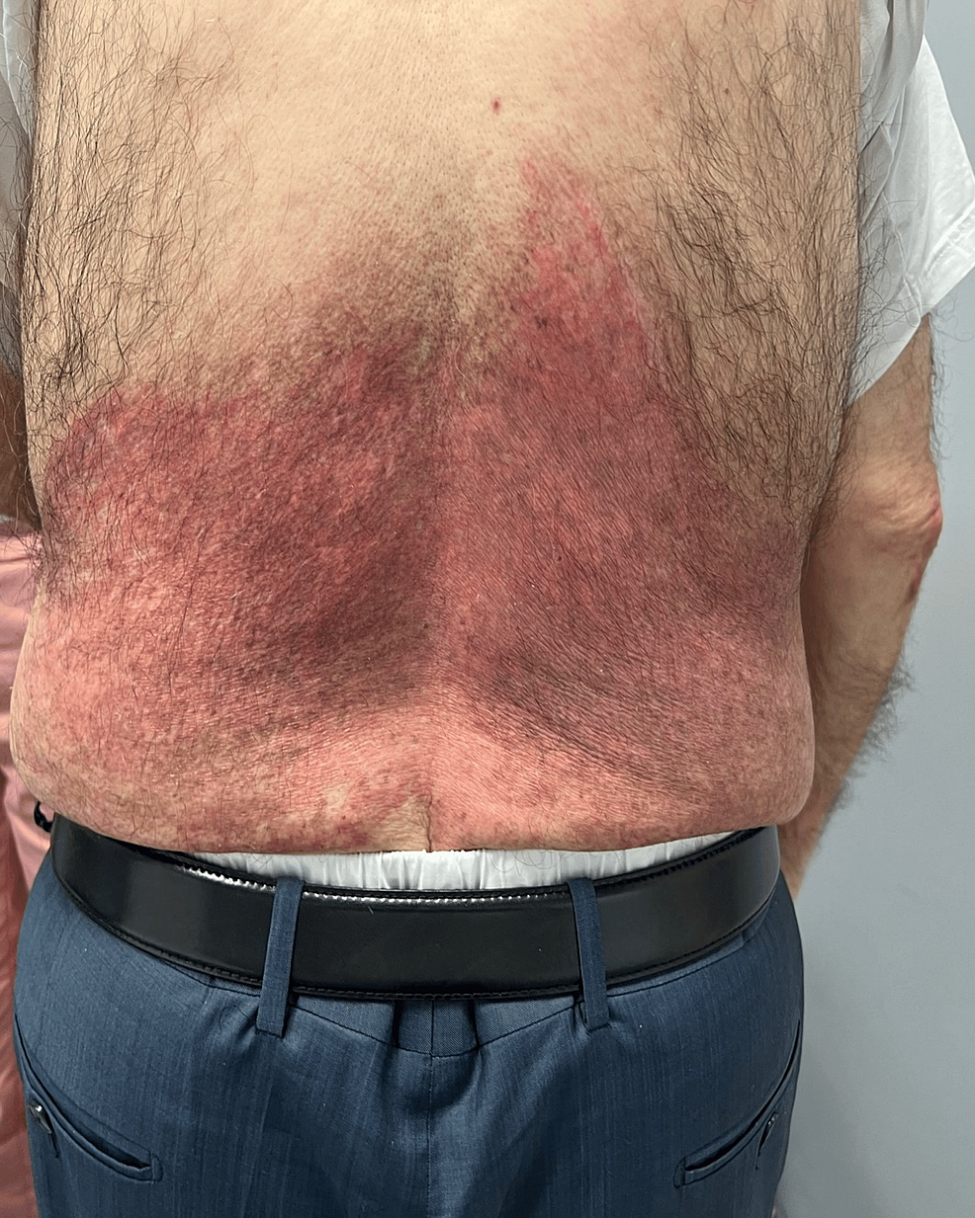
Erythematous scaly plaques over the lower back a few weeks after initiation of treatment with hydroxychloroquine

Based on the strongly positive Ro and La antibodies, we believe our patient has Sjogren’s syndrome, in spite of the fact he had no dryness in the eyes or mouth. He was treated with hydroxychloroquine 200 mg twice daily and corticosteroids were gradually tapered. A follow-up visit a few weeks after initiating treatment demonstrated gradual resolution of the erythematous lesions (Figures 6, 7).

## Discussion

Among connective tissue diseases, vacuolar interface dermatitis has been mainly linked to lupus erythematosus and dermatomyositis. In both conditions, dermatitis appears as one or more scaling plaques in photodistributed areas, typically involving the head and neck region [5] but other parts of the skin may also be affected leading to specific and non-specific lesions over the torso and the extremities. Variable degree of dermal mucinosis and vascular alterations that vary according to the age are additional microscopic findings frequently observed in these two conditions [5].

Keratinocyte damage in SS has been attributed to the surface binding of anti-Ro antibodies that may allow adherence of membrane attack complex of complement (C5b-9) resulting in formation of plasmalemmal pores. In concert with antibody-dependent cellular cytotoxicity, it might contribute to keratinocyte injury [6]. Vascular deposition has been observed in the setting of SLE with positive antibodies to Ro [7].

Since early diagnosis and appropriate treatment are essential for optimal management of SS, it is vital to recognize the presenting symptoms and signs. Extreme fatigue is a common symptom among patients with SS. It can affect approximately 50% of the SS population, and may often be the presenting symptom of SS [8]. Arthralgias and myalgias are also common among the SS population [9]. Patients might present with non-erosive arthritis, resembling that of SLE [10]. A new-onset cough can be an initial manifestation of SS representing xerotrachea [11].

Although xerostomia and xerophtalmia are common complaints of patients with SS, other skin manifestations may be present [12]. These cutaneous manifestations are thought to be mediated by B-cell hyperactivity [13]. Examples include eyelid dermatitis and cutaneous dermatitis [12].

Certain medications may precipitate SS; these include dopamine agonists, antiretroviral therapy, calcium channel blockers, and beta-blockers are the commonest medications contributing to SS symptoms [14]. It might be plausible that our patient developed medication-induced SS, although the symptoms did not resolve after discontinuing the possible triggering medications.

In addition to wound care, patient education, smoking cessation, and multiple pharmacological options are used for the treatment of the cutaneous manifestations of SS. Topical treatments, such as topical corticosteroids, may be used as a symptomatic and initial treatment [13]. Locally applied omega-3 essential fatty acids have anti-inflammatory properties and may improve the quality of life [15]. As for systemic treatments, typical SS treatments are also effective for cutaneous manifestations. Hydroxychloroquine, belimumab, and abatacept have shown positive results [12].

## Conclusions

Apart from the widely known SS and medication-induced SS manifestations, specifically xerostomia and xerophtalmia, certain dermatological manifestations may be the initial presenting symptoms. Interface vacuolar dermatitis has been linked to certain connective tissue diseases, most commonly dermatomyositis and SLE. While vacuolar degeneration has been linked to anti-Ro and anti-La antibodies, the evidence regarding the existence of interface vacuolar dermatitis in the context of SS is still not clear. While systemic treatments for SS are essential to help in resolution of the dermatological manifestations, local care is also important for quality of life improvement.
